# Imbalance of Th17, Treg, and helper innate lymphoid cell in the peripheral blood of patients with rheumatoid arthritis

**DOI:** 10.1007/s10067-022-06315-8

**Published:** 2022-08-04

**Authors:** Ting Wang, Jinbing Rui, Wenqi Shan, Fei Xue, Dingqi Feng, Liyang Dong, Jiahui Mao, Yang Shu, Chaoming Mao, Xuefeng Wang

**Affiliations:** 1 Department of Central Laboratory, The Affiliated Hospital of Jiangsu University, 438 Jiefang Road, Zhenjiang, 212001 China; 2 Department of Clinical Laboratory, The Affiliated Hospital of Jiangsu University, Zhenjiang, 212001 China; 3 Department of Rheumatology, The Affiliated Hospital of Jiangsu University, Zhenjiang, 212001 China; 4 Department of Nuclear Medicine and Institute of Oncology, The Affiliated Hospital of Jiangsu University, Zhenjiang, 212001 China

**Keywords:** Helper innate lymphoid cell, Rheumatoid arthritis, Th17, Treg

## Abstract

**Introduction:**

Rheumatoid arthritis (RA) is a chronic inflammatory disease involving a variety of immune cells, including adaptive T and B cells and innate lymphoid cells (ILCs). Understanding the pathogenic role of these immune cells in RA provides new insights into the intervention and treatment of RA.

**Methods:**

A total of 86 patients with RA (RA group) and 50 healthy controls (HC) were included in the study. The immune cells of CD4^+^, CD19^+^ B, NK, Th17, Treg, ILCs, and their subsets (i.e., ILC1s, ILC2s, and ILC3s) were characterized in peripheral blood mononuclear cells by flow cytometry. Cytokines (i.e., IFN-γ, IL-4, IL-10, IL-17A, IL-22, and IL-33) in sera were detected using ELISA. The above immune cells and cytokines were analyzed in patients with different disease activity status and positive ( +) or negative ( −) rheumatoid factor (RF)/anti-citrullinated protein antibodies (ACPA).

**Results:**

Patients with RA had higher percentages of CD4^+^ T, CD19^+^ B, Th17, ILC2s, and ILC3s and lower percentages of Treg and ILC1s than HC. Patients with RA had elevated levels of IFN-γ, IL-4, IL-17A, and IL-22 and decreased level of IL-10. Compared with HC, patients with high disease activity had higher percentages of Th17, ILC2s, and ILC3s; lower percentages of ILC1s; and lower level of IL-10. The percentage of Treg cells in remission, low, moderate, and high disease activities decreased, whereas the level of IL-17A increased compared with HC. Furthermore, RF^+^ or ACPA^+^ patients exhibited elevated percentages of CD19^+^ B, ILC2s, and ILC3s and had decreased percentage of ILC1s and Treg cells than HC. The percentage of Th17 cells increased in RF^−^/ACPA^−^ and RF^+^/ACPA^+^ patients. However, the above immune cells between RF or ACPA positive and negative patients were not significantly different.

**Conclusion:**

Th17, Treg, and ILC subset dysregulations are present in patients with RA but may not be associated with conventionally defined seropositive RF and ACPA.

**Table:** 

**Key Points** *• Th17, Treg, and ILC subset dysregulations are present in patients with RA but may reflect inflammation rather than specific diseases and stages.* *• No difference for the distribution of Th17, Treg, and ILC subsets between RF*^+^ *and RF*^*−*^* patients and between ACPA*^+^ *and ACPA*^*−*^* patients. The screening spectrum of RF and ACPA serology should be expanded to elucidate the role of immune cells in RA pathogenesis.*

**Supplementary Information:**

The online version contains supplementary material available at 10.1007/s10067-022-06315-8.

## Introduction

Rheumatoid arthritis (RA) is a chronic inflammation characterized by the infiltration of inflammatory cells in the joint synovium, causing joint destruction, disability, and pathological change of extra-articular sites. Recently, the pathogenesis of RA demonstrated that the aberrant activation of innate and adaptive immune cells by the complex interaction between genetic and environmental factors, which disrupts the immune tolerance, causes the presentation of autoantigen and inflammatory cytokine secretion [1, 2]. Various immune cells have been found in the joint of patients with RA, especially CD4^+^ T cells, B cells, and macrophages [3, 4]. Cytokines produced by macrophage activate fibroblast-like synoviocytes and osteoclasts, promote bone destruction, and drive CD4^+^ T cell polarization and B cell activation [3, 5]. Th1 cells are originally thought to be one of the main pathogenic factors of RA. Recently, IL-17 and Th17 cells have been thought to have critical roles in RA pathogenesis. CD14^+^ monocytes from the inflamed joints of patients with RA can potently induce Th17 cells instead of Th1 cells [6]. Furthermore, B cells/plasma cells produce autoantibodies especially rheumatoid factors (RF) and anti-citrullinated peptide antibodies (ACPAs), which promote T cell activation [7, 8]. Thus, autoreactive T and B cells play a critical role in the pathogenesis of RA.

In addition to B and T cells, innate lymphoid cells (ILCs) are considered to play an important role in the pathogenesis of RA. Helper-like lymphoid cells, as the most prominent ILCs, are usually classified into ILC1, ILC2, and ILC3 subsets and are counterpart of Th cells [9]. Similar to Th cells, ILC1s require T-bet and produce IFN-γ. ILC2s express GATA3 and secrete IL-4, IL-5, and IL-13. ILC3s express RORγt and secrete IL-17 and IL-22. ILCs can be a bridge between innate and adaptive immunity, thereby mediating inflammatory responses, including RA [9]. Takaki-Kuwahara and colleagues reported that CCR6^+^ILC3s increases in joints of arthritic mice and patients with RA, and CCR6^+^ILC3s may participate in the development of RA by producing IL-17 and IL-22 [10]. However, Yang and colleagues found that stable patients with RA have increased proportions of ILC2s and reduced percentages of ILC1s and ILC3s. The percentages of ILC1s and ILC2s are increased in mice with collagen-induced arthritis (CIA), indicating that the dysregulation of ILCs participates in the development of RA and CIA [11]. Thus, we hypothesized that intrinsic and acquired immune cell imbalances, accompanied with the production of inflammatory cytokines, are associated with the development of RA. An improved understanding of the role and function of the abovementioned innate and adaptive immune cells, especially ILC subsets, in the pathogenesis of RA can provide new interventions for the treatment of RA.

In this study, we analyze the immune cells especially ILCs and their three subsets in the peripheral blood of patients with RA by flow cytometry. We demonstrate that the proportions of CD4^+^T, CD19^+^B, Th17, ILC2, and ILC3 cells in the peripheral blood of patients with RA increase, and the proportions of Treg and ILC1 cells decrease. The percentages of CD19^+^ B, Th17, ILC2s, and ILC3s increase in RF- (RF^+^) or ACPA-positive (ACPA^+^) patients. The percentages of ILC1s and Treg cells decrease in RF^+^ or ACPA^+^ patients. The above immune cells have not been found with a significant difference between RF or ACPA seropositive and seronegative patients, suggesting that the dysregulation of these immune cells may not be associated with conventionally defined seropositive RF and ACPA.

## Materials and methods

### Patients

A total of 86 patients with RA fulfilling the criteria of the American College of Rheumatology [12] and 50 age- and sex-matched healthy controls were enrolled into this study. Disease activity was assessed using the 28-joint disease activity score (DAS28) [13], and all patients were free of infectious diseases, malignant diseases, cardiovascular complaints, or other inflammatory diseases. The characteristics of patients with RA and healthy controls are summarized in Table S11. None had been taking disease modifying anti-rheumatic drugs. On the basis of DAS28 values, patients were classified as remission (DAS28 < 2.6, *n* = 25), low-disease activity (2.6 ≤ DAS28 ≤ 3.2, *n* = 19), moderate-disease activity (3.2 < DAS28 ≤ 5.1, *n* = 27), and high-disease activity (DAS28 > 5.1, *n* = 15) groups as previously described [14]. Informed consent was obtained from all patients and healthy controls, and the study was approved by the ethics committee of the Affiliated Hospital of Jiangsu University (Permit Number: SWYXLL20191119-15) and performed in accordance with the Helsinki Declaration on ethical principles for medical research involving human participants.

### Blood sample preparation

Peripheral blood mononuclear cells (PBMC) were isolated from patients with RA and HC by the Ficoll–Hypaque (Solarbio, Beijing, China) density gradient centrifugation. Then, PBMC were performed immunophenotyping by flow cytometry. Serum was separated from the specimens and stored at − 80 °C until use for cytokine determination by enzyme-linked immunosorbent assay (ELISA). ACPA was measured using an anti-cyclic citrullinated peptide assay ELISA kit (Fuchunkexin, Shanghai, China) with an upper limit of normal of 25 IU/mL. ACPA > 25 IU/mL was considered positive (ACPA^+^), and ACPA < 25 IU/mL was considered negative (ACPA^−^) as previously described [15]. The C-reactive protein (CRP) and IgM-RF were measured using the Beckman Coulter IMMAGE800 (Beckman Coulter). RF > 20 IU/mL was considered positive (RF^+^), whereas RF < 20 IU/mL was considered negative (RF^−^) [15].

### Flow cytometry

For the analysis of Th17 cells, PBMC were suspended at a density of 1 × 10^6^ cells/mL in complete culture medium (RPMI-1640 supplemented with 10% heat-inactivated fetal calf serum) for 5 h in the presence of phorbol myristate acetate (25 ng/mL) and brefeldin A (1 μg/mL) at 37 °C and 5% CO_2_. Cells were then incubated with human fluorescein isothiocyanate (FITC)–anti-CD4 mAb, washed, fixed, and permeabilized with Cytofix/Cytoperm (BD PharMingen, San Diego, CA, USA). Cells were then intracellularly stained with phycoerythrin (PE)–anti-IL-17A or PE-conjugated rat IgG1 (isotype control) for 30 min at 4 °C based on previous publications [16].

For the analysis of Treg cells, PBMC were stained with human FITC–anti-CD4 mAb, and APC–anti-CD25 mAb, fixed, permeabilized with Cytofix/Cytoperm, and intracellularly stained with PE–anti-Foxp3 or PE–IgG2a rat IgG control antibody in accordance with the manufacturer’s instructions.

For the analysis of ILCs, the following antibodies were used to quantify ILCs based on previous publications [17]: eFluor450-conjugated anti-CD3 (eBioscience), allophycocyanin (APC)–Cyanine7-conjugated anti-CD19 (eBioscience), PE–Cyanine7-conjugated anti-CD56 (NCAM, eBioscience), FITC-conjugated anti-CD127 (eBioscience), APC-conjugated anti-CD117 (cKit, eBioscience), and PE-conjugated anti-CD294 (CRTH2, eBioscience). ILCs were defined as cells within the lymphocyte gate on the scatter plot that were single cells, lineage negative (i.e., CD3^−^CD19^−^CD56^−^) and CD127 positive. ILCs were subsequently classified as ILC1s (Lin^−^CD127^+^CRTH2^−^c-Kit^−^), ILC2s (Lin^−^CD127^+^CRTH2^+^), or ILC3s (Lin^−^CD127^+^CRTH2^−^c-Kit^+^) based on the markers CD117 (c-Kit) and CD294 (CRTH2). Samples were analyzed using the BD FACSCanto flow cytometer (BD Biosciences) and Flowjo Software (Tree Star).

### ELISA

The analysis of cytokines in serum from patients with RA and HC was conducted using human ELISA kits (Multi Sciences, Hangzhou, China). All assays were performed in accordance with the manufacturer’s instructions. Six cytokines, i.e., human interferon (IFN)-γ, interleukin (IL)-4, IL-10, IL-17A, IL-22, and IL-33, were measured.

### Statistical analyses

Normally distributed data are presented as mean ± SEM. Student’s t-test or one-way ANOVA test were used for determining significant differences between groups. Non-normally distributed data were presented as median (interquartile range) and analyzed using the Mann–Whitney U or Kruskal–Wallis test. *P* values < 0.05 were considered significant. Data were analyzed using the GraphPad Prism version 8.0.

## Results

### Elevation of circulating CD4^+^ T, CD19^+^ B, Th17, ILC2s, and ILC3s percentages and reduction of Treg and ILC1s percentages in patients with RA

**Fig. 1:**
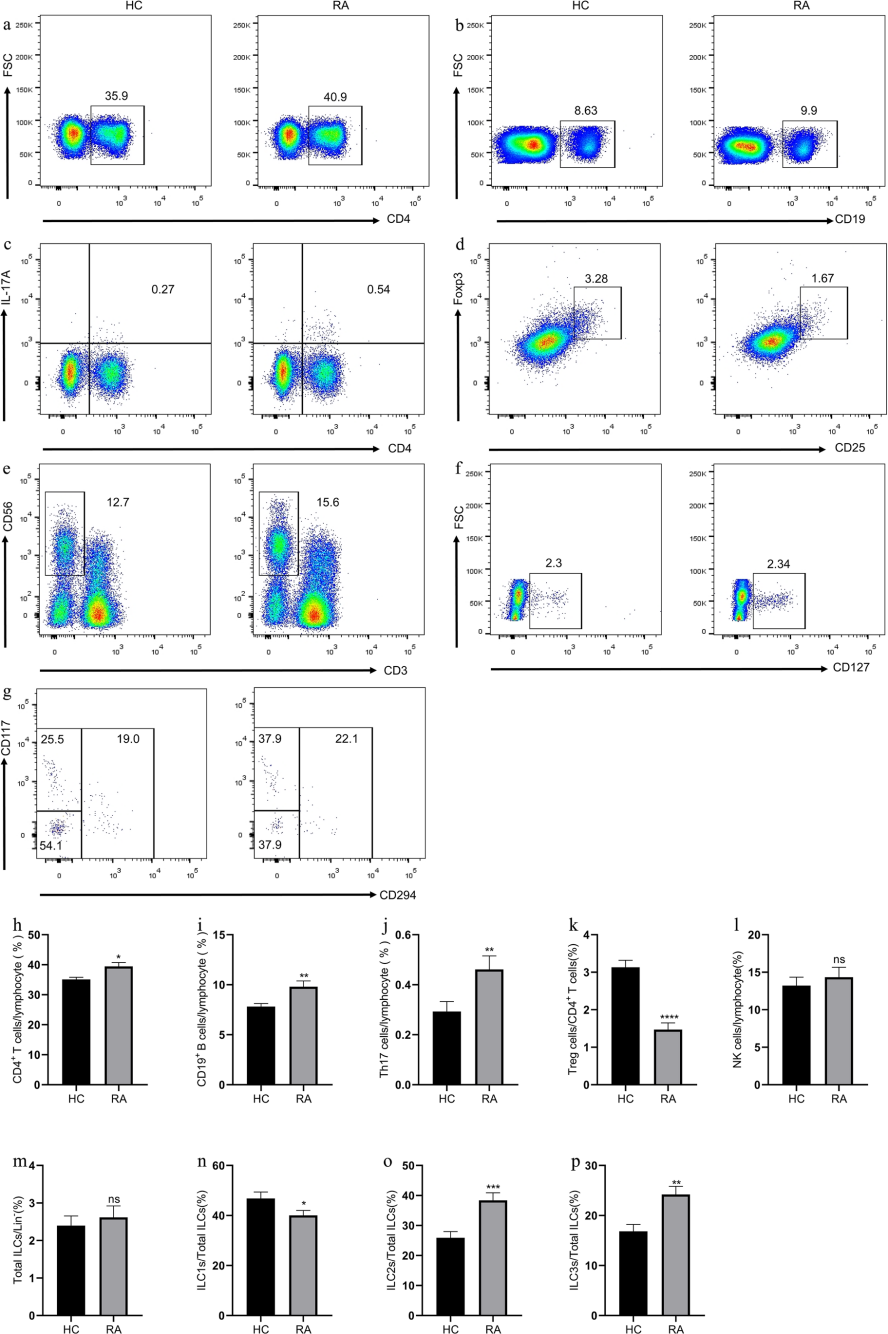
Flow cytometry analysis of circulating lymphocyte subsets in RA patients and HC. **a** CD4^+^ T cells, **b** CD19^+^ B cells, **c** CD4^+^IL-17^+^ Th17 cells, **d** CD4^+^CD25^+^Foxp3^+^ Treg cells, **e** CD3^−^CD56^+^ NK cells, **f** CD3^−^CD19^−^CD56^−^CD127^+^ ILCs, and **g** Lin^−^CD294^−^CD117^−^ ILC1s, Lin^−^CD294^+^ ILC2s, and Lin^−^CD294^−^CD117^+^ ILC3s. Data are representative of the experiments. **h** The percentage of CD4^+^ T cells, **i** CD19^+^ B cells, **j** Th17 cells, **k** Treg cells, **l** NK cells, **m** total ILCs, **n** ILC1s, **o** ILC2s, and **p** ILC3s in each group. Results are presented as mean ± SEM. *P < 0.05,**P < 0.01, ***P < 0.001

Blood lymphocytes from patients with RA and HC were analyzed by flow cytometry. Consistent with the participation of CD4^+^T and B cells in the pathogenesis of RA [18], the percentages of CD4^+^ T and CD19^+^ B cells in patients with RA were remarkably increased compared with those in HC (Fig. 1). Furthermore, consistent with our results and others described previously [19, 20], compared with those in HC, the percentage of Th17 cells (0.46 ± 0.05, *P* = 0.0025) in patients with RA increased, whereas the percentage of Treg cells (1.47 ± 0.18, *P* < 0.0001) decreased (Fig. 1). No significant difference was observed between NK cells (14.35 ± 1.32, *P* = 0.7725) and total ILCs (2.62 ± 0.30, *P* = 0.89) between patients with RA and HC (Figure S1 for gating strategy of ILCs). However, patients with RA had increased percentages of ILC2s (38.41 ± 2.48, *P* = 0.0002) and ILC3s (24.22 ± 1.62, *P* = 0.0018) and decreased percentage of ILC1s (40.09 ± 1.93, *P* = 0.037) compared with HC (Fig. 1). Thus, B and CD4^+^T cells especially Th17/Treg and ILC subsets imbalance contributed to the development of the inflammatory process of RA.

### Elevation of IFN-γ, IL-4, IL-17A, and IL-22 levels and reduction of IL-10 level in patients with RA

**Fig. 2:**
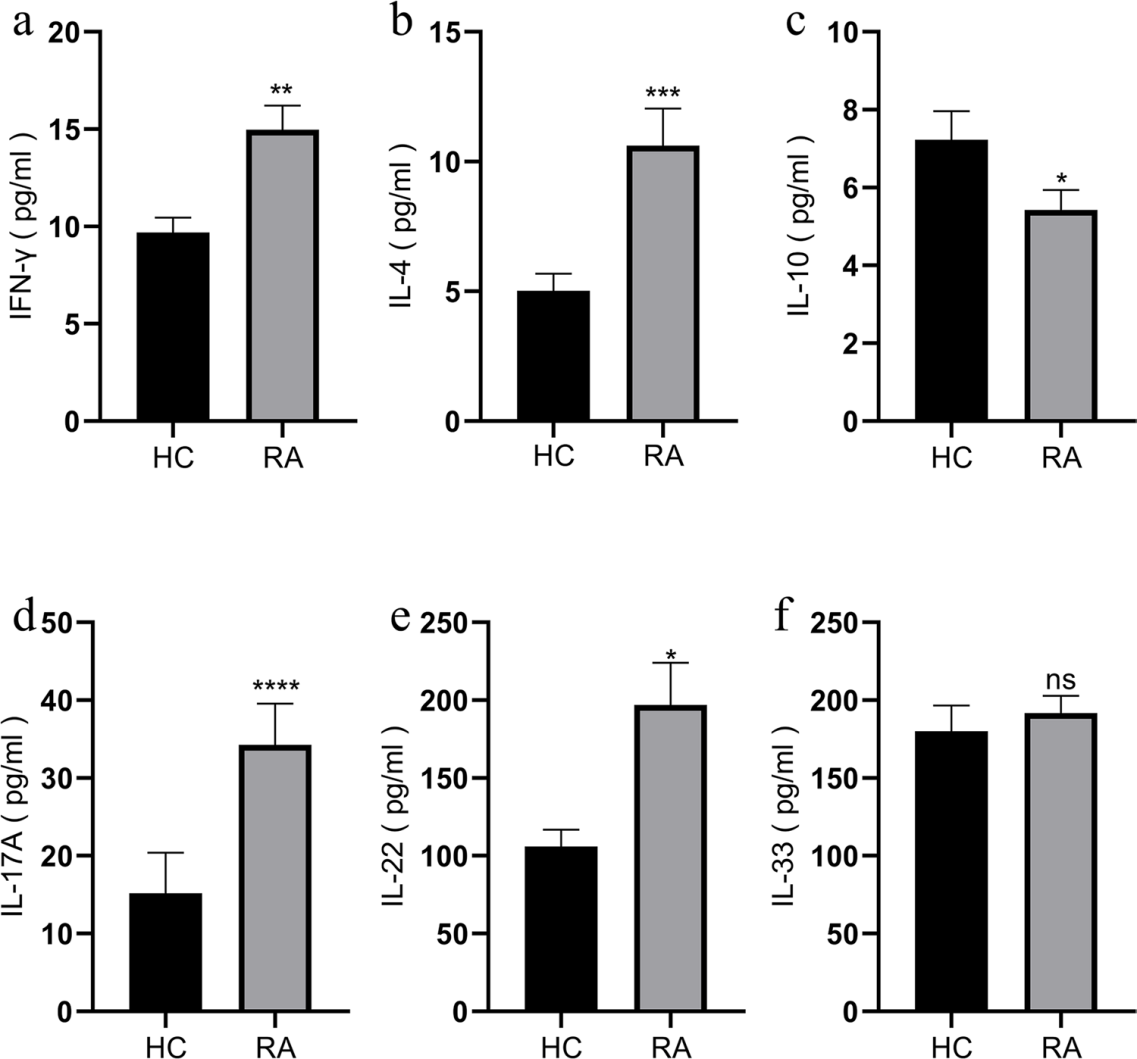
Cytokine analysis in sera by ELISA in RA patients and HC. **a** IFN-γ, **b** IL-4, **c** IL-10, **d** IL-17A, **e** IL-22, and **f** IL-33. Results are presented as mean ± SEM. *P < 0.05, ***P < 0.001, ****P < 0.0001

Considering the important role of cytokines in the pathogenesis of RA, we analyzed the cytokine level in the sera of patients with RA and HC. As shown in Fig. 2, compared with that of HC, the sera of patients with RA was observed with higher levels of IFN-γ (14.98 ± 1.22, *P* = 0.0042), IL-4 (10.62 ± 1.43, *P* = 0.0006), IL-17A (34.27 ± 5.30, *P* < 0.0001), and IL-22 (197.00 ± 27.13, *P* = 0.0483) and lower level of IL-10 (5.43 ± 0.50, *P* = 0.043). However, the level of IL-33 (191.90 ± 10.96, *P* = 0.4814) between patients with RA and HC was not statistically different (Fig. 2).

### Elevation of circulating Th17, ILC2s, and ILC3s percentages and reduction of Treg and ILC1s percentages in patients with RA and high disease status

**Fig. 3:**
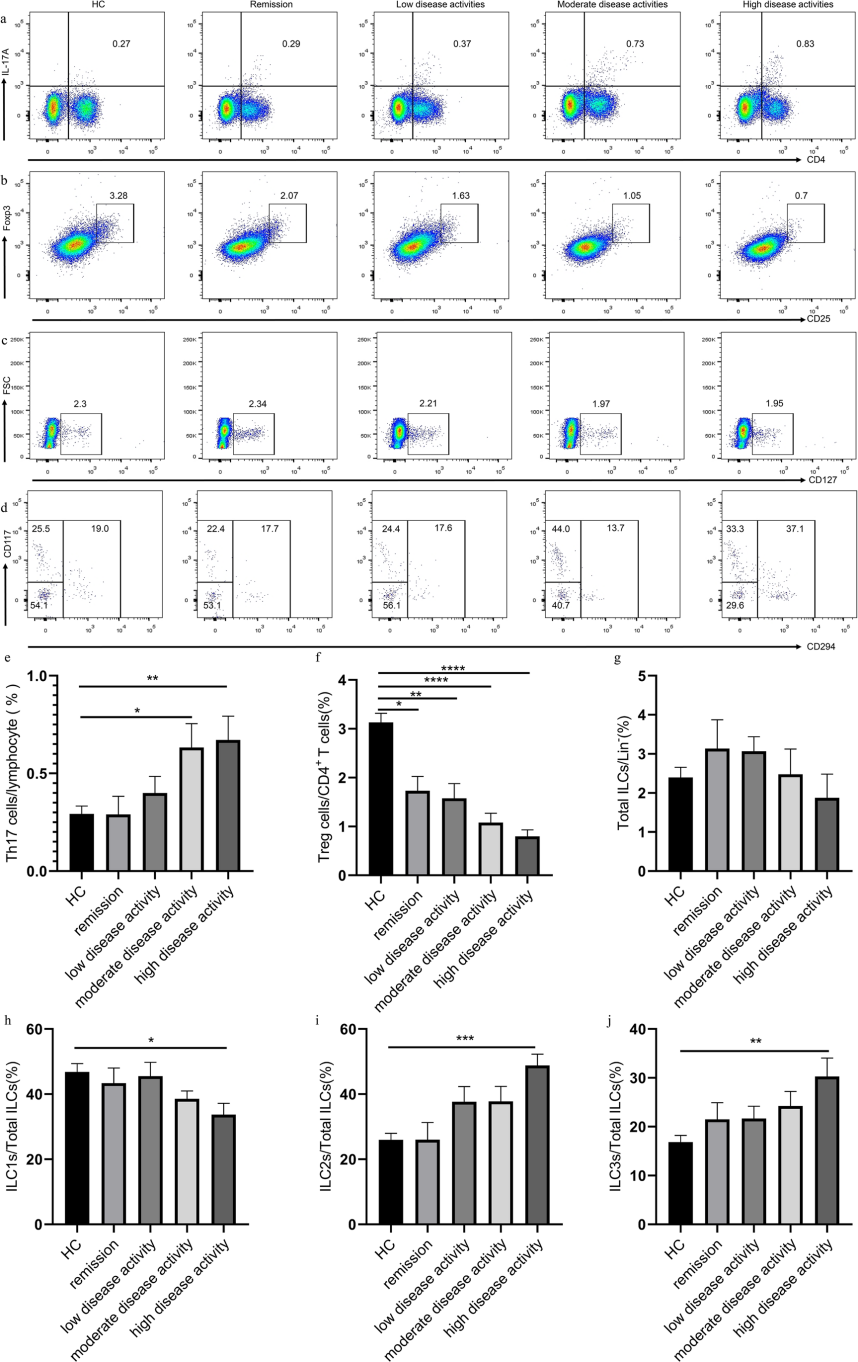
Flow cytometry analysis of circulating lymphocyte subsets in HC and RA patients with different disease activity status. **a** CD4^+^IL-17^+^ Th17 cells, **b** CD4^+^CD25^+^Foxp3^+^ Treg cells, **c** CD3^−^CD19^−^CD56^−^CD127^+^ ILCs, and **d** Lin^−^CD294^−^CD117^−^ ILC1s, Lin^−^CD294^+^ ILC2s, and Lin^−^CD294^−^CD117^+^ ILC3s. Data are representative of the experiments. **e** The percentage of Th17 cells, **f** Treg cells, **g** total ILCs, **h** ILC1s, **i** ILC2s, and **j** ILC3s in each group. Results are presented as mean ± SEM. *P < 0.05, **P < 0.01, ***P < 0.001,****P < 0.0001

To explore the relationship between circulating immune cells and disease activity, we analyzed the immune cell phenotype in different disease activity status of patients with RA. The clinical characteristics of different disease activity status are shown in Figure S2. Compared with the HC, groups with low (30.74 ± 3.47, *P* = 0.0002), moderate (54.56 ± 6.69, *P* < 0.0001), and high disease activities displayed higher ESR (77.00 ± 17.43, *P* < 0.0001). Groups with low (30.74 ± 3.47, *P* = 0.0490), moderate (54.56 ± 6.69, *P* < 0.0001), and high disease activities (77.00 ± 17.43, *P* = 0.0082) also showed higher ESR level compared with the remission group. CRP (12.46 ± 1.80, *P* < 0.0001) and RF (192.9 ± 35.58, *P* < 0.0001) levels were higher in patients with RA regardless of disease activity status compared with those in HC (Figure S2). No statistical difference in CRP and RF levels was observed among remission group and different disease activity groups. However, the percentages of Th17 cells in moderate- (0.63 ± 0.12, *P* = 0.0138) and high-disease activity groups (0.67 ± 0.12, *P* = 0.0028) were significantly increased compared with those in the HC (Fig. 3). Furthermore, the high-disease activity group had higher levels of ILC2s (48.82 ± 3.47, *P* < 0.0001) and ILC3s (30.30 ± 3.76, *P* = 0.0249) and lower level of ILC1s (33.71 ± 3.46, *P* = 0.0346) compared with HC (Fig. 3). The percentage of Treg cells in remission (1.73 ± 0.30, *P* = 0.0104), low (1.58 ± 0.30, *P* = 0.0034), moderate (1.08 ± 0.19, *P* < 0.0001), and high disease activities (0.80 ± 0.13, P < 0.0001) also decreased compared with HC (Fig. 3). No significant difference in the proportion of total ILCs (2.62 ± 0.30, *P* = 0.89) was observed among the healthy control group and different disease activity groups. The distributions of Th17, Treg, and ILC subsets were not different among the different disease stages.

### Elevated IL-17A level and decreased IL-10 level in the serum of patients with high disease activity

**Fig. 4:**
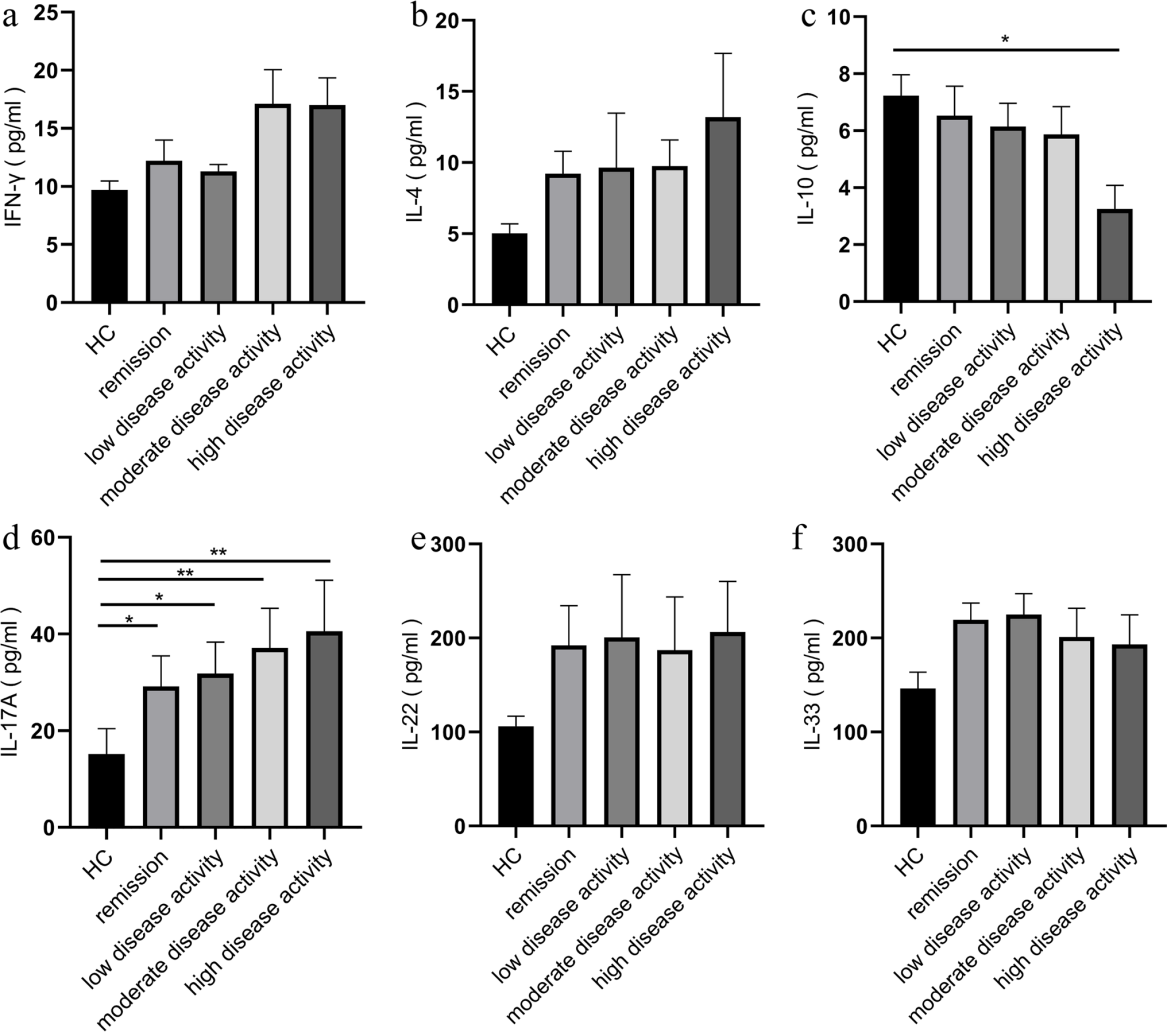
Cytokine analysis in sera by ELISA in HC and RA patients with different disease activity status. **a** IFN-γ, **b** IL-4, **c** IL-10, **d** IL-17A, **e** IL-22, and **f** IL-33. Results are presented as mean ± SEM. *P < 0.05, **P < 0.01

Given that cytokines are the key inflammatory mediators in patients with RA, we analyzed the cytokine secretion in sera from different disease activity status of patients with RA. As shown in Fig. 4, compared with healthy controls, patients in the remission group (29.17 ± 6.32, *P* = 0.0112) and low- (31.82 ± 6.50, *P* = 0.0118), moderate- (37.14 ± 8.17, *P* = 0.0020), and high-disease activity groups (40.59 ± 10.55, *P* = 0.0032) had increased IL-17A level. However, the IL-10 level (3.26 ± 0.82, *P* = 0.0233) was decreased in patients with high disease activity. Furthermore, the levels of IFN-γ, IL-4, IL-22, and IL-33 were not statistically different among HC and patients with RA and different disease activity status.

### Elevation of CD19^+^ B, Th17, ILC2s, and ILC3s percentages and reduction of Treg and ILC1s percentages in RF^+^ patients

**Fig. 5:**
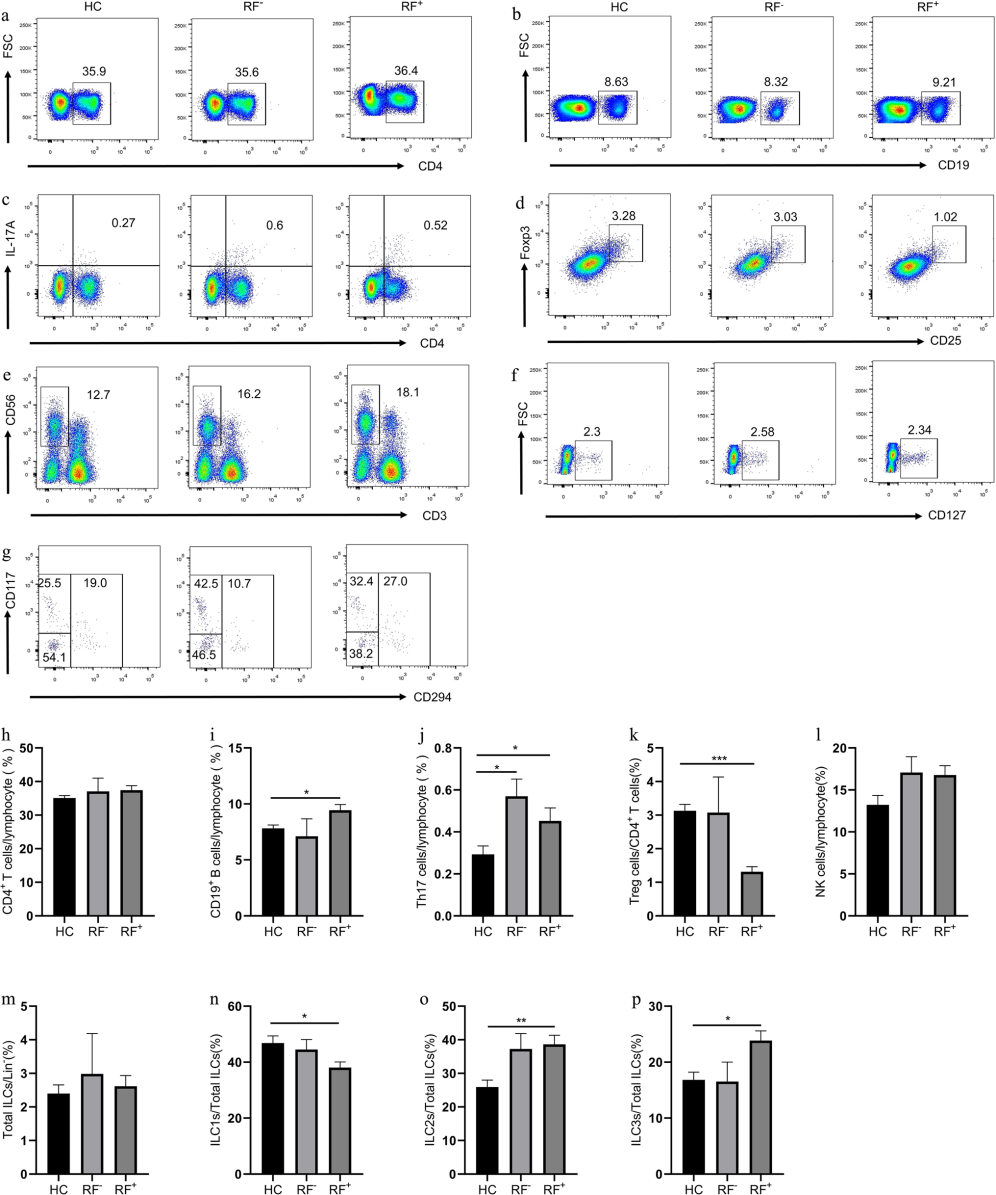
Flow cytometry analysis of circulating lymphocyte subsets in HC, RF^−^, and RF^+^ RA patients. **a** CD4^+^ T cells, **b** CD19^+^ B cells, **c** CD4^+^IL-17^+^ Th17 cells, **d** CD4^+^CD25^+^Foxp3^+^ Treg cells, **e** CD3^−^CD56^+^ NK cells, **f** CD3^−^CD19^−^CD56^−^CD127^+^ ILCs, and **g** Lin^−^CD294^−^CD117^−^ ILC1s, Lin^−^CD294^+^ ILC2s, and Lin^−^CD294^−^CD117^+^ ILC3s. Data are representative of the experiments. **h** The percentage of CD4^+^ T cells, **i** CD19^+^ B cells, **j** Th17 cells, **k** Treg cells, **l** NK cells, **m** total ILCs, **n** ILC1s, **o** ILC2s, and **p** ILC3s in each group. Results are presented as mean ± SEM. *P < 0.05, **P < 0.01, ***P < 0.001

RF is the first autoantibody and is related to high disease activity [21]. Thus, we analyzed the immune cell phenotype and cytokine secretion in RF^+^ and RF^−^ patients. As shown in Fig. 5, RF^+^ patients had increased percentages of CD19^+^ B (9.44 ± 0.50, *P* = 0.0354), ILC2s (38.64 ± 2.72, *P* = 0.0009), and ILC3s (23.87 ± 1.72, *P* = 0.0352) and decreased percentage of Treg (1.32 ± 0.15, *P* < 0.0001) and ILC1s (38.06 ± 2.02, *P* = 0.0396) compared with HC (Fig. 5). RF^+^ (0.45 ± 0.06, *P* = 0.0318) and RF^−^ (0.57 ± 0.08, *P* = 0.0164) patients had increased Th17 cell percentage, and the levels of IL-17A of RF^+^ (50.21 ± 9.73, *P* =  < 0.0001) and RF^−^ (32.98 ± 6.11, *P* = 0.0392) patients were higher compared with HC. However, no significant difference in the proportion of CD4^+^ T, Treg, NK, and total ILCs was observed among the healthy control, RF^+^, and RF^−^ groups. The levels of IFN-γ, IL-4, IL-10, IL-22, and IL-33 were not statistically different among HC and RF^+^ or RF^−^ patients (Figure S3). Furthermore, the above cells and cytokines displayed no significant difference between RF^+^ and RF^−^ patients.

### Elevation of CD19^+^ B, Th17, ILC2s, and ILC3s percentages and reduction of Treg and ILC1s percentages in ACPA^+^ patients

**Fig. 6:**
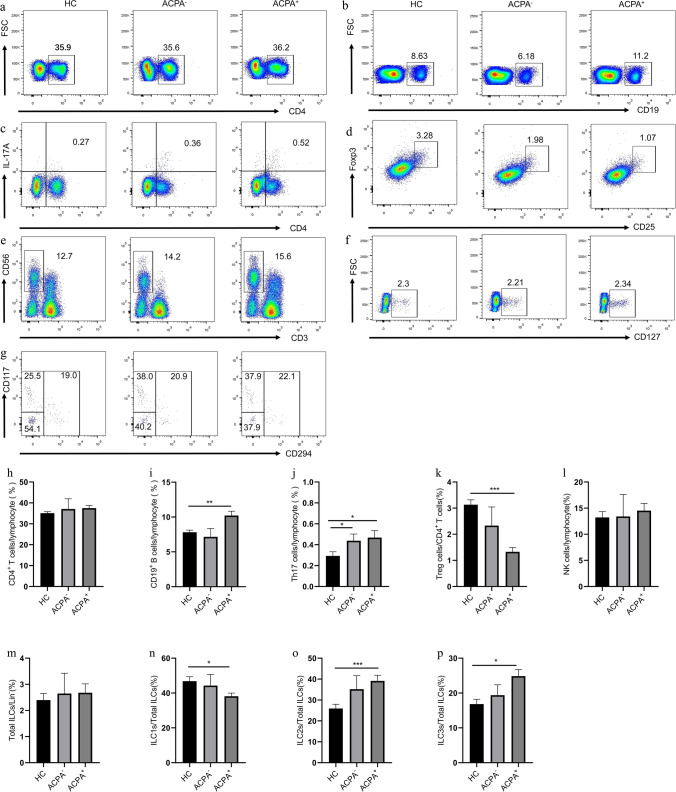
Flow cytometry analysis of circulating lymphocyte subsets in HC, ACPA^−^, and ACPA^+^ RA patients. **a** CD4^+^ T cells, **b** CD19^+^ B cells, **c** CD4^+^IL-17^+^ Th17 cells, **d** CD4^+^CD25^+^Foxp3^+^ Treg cells, **e** CD3^−^CD56^+^ NK cells, **f** CD3^−^CD19^−^CD56^−^CD127^+^ ILCs, and **g** Lin^−^CD294^−^CD117^−^ ILC1s, Lin^−^CD294^+^ ILC2s, and Lin^−^CD294^−^CD117^+^ ILC3s. Data are representative of the experiments. **h** The percentage of CD4^+^ T cells, **i** CD19^+^ B cells, **j** Th17 cells, **k** Treg cells, **l** NK cells, **m** total ILCs, **n** ILC1s, **o** ILC2s, and **p** ILC3s in each group. Results are presented as mean ± SEM. *P < 0.05, **P < 0.01, ***P < 0.001

ACPA is another main antibody and is related to the disease progression of patients with RA [22, 23]. Thus, the immune cell phenotype and cytokine secretion in ACPA^+^ and ACPA^−^ patients were analyzed. As shown in Fig. 6, ACPA^+^ patients showed increase percentages of CD19^+^ B (10.21 ± 0.61, *P* = 0.0060), ILC2s (39.15 ± 2.70, *P* = 0.0445), and ILC3s (24.86 ± 1.89, *P* = 0.0130) and decreased percentages of Treg (1.33 ± 0.16, *P* < 0.0001) and ILC1s (38.16 ± 1.84, *P* = 0.0391) compared with HC (Fig. 6). ACPA^+^ (0.47 ± 0.07, *P* = 0.0445) and ACPA^−^ patients (0.44 ± 0.06, *P* = 0.0364) all displayed the increased percentage of Th17 cells compared with HC. Serum IL-17A in ACPA^+^ (33.29 ± 3.75, *P* < 0.0001) and ACPA^−^ (32.20 ± 6.47, *P* = 0.0408) patients were higher than those in HC (Figure S4).

## Discussion

RA is a chronic inflammatory disease. Although the pathogenic mechanisms of RA have not been fully elucidated, the aberrant activation of innate and adaptive immune system with consequent production of autoantibodies and inflammatory cytokines participates in the development of RA [18]. In this setting, various immune cells, such as T cells, B cells, and other innate immune cells, form a complex network and produce proinflammatory cytokines, which eventually lead to joint damage [3]. In addition to adaptive T and B cells, the ILCs involved in inflammatory and autoimmune responses have been confirmed to participate in the pathogenesis of RA [3]. Rauber and colleagues demonstrated that patients with RA and in remission have high numbers of IL-9^+^ILC2s [24]. In the present study, we demonstrated that patients with RA have upregulated percentages of circulating CD4^+^ T, CD19^+^ B, Th17, ILC2s, and ILC3s and downregulated percentages of Treg and ILC1s. Increased Th17 and decreased Treg frequencies in patients with RA in the present study are consistent with previous studies [19, 20]. In contrast to the reduction of ILC1s and increase of ILC3s in PBMC of patients with RA, Leijten and colleagues reported that the frequencies of ILC1s increase, and ILC3s decrease in the synovial fluid (SF) of patients with RA [25]. ILCs initially serve as tissue “sentinel,” which can produce a variety of cytokines upon activation in an inflammatory environment. Furthermore, ILCs initiate helper T cell responses [26], and activated Th cells produce proinflammatory cytokines in chronic inflammation. Consistent with increased ILC2s and ILC3s frequencies, IL-4 and IL-17 levels in the sera of patients with RA are also increased. However, whether IL-4 is secreted from ILC2s or Th2 and whether IL-17 is produced from ILC3s or Th17 are unclear. Inconsistent with the decrease in ILC1s percentage, the IFN-γ level in the sera of patients with RA is increased. Th1 cells and IFN-γ have been suggested to contribute to the pathogenesis in RA[27], and IL-4 and other Th2 cytokines downregulate the inflammatory processes in RA [28, 29]. However, SF from patients with early arthritis display increased Th2 cytokines, such as IL-4 and IL-13, but not Th1-related IFN-γ [30]. Furthermore, Kokkonen and colleagues reported that the sera from patients after the development of RA show increases in Th1-related IFN-γ, Th2-related IL-4 and IL-13, and immune regulation IL-10 [31]. In addition, IFN-γ may be derived from Th17 and CD8^+^ T cells (Tc1 cells) in patients with RA [27, 32] although we have not identified IFN-γ-producing cells. Consistent with the increase in Th17 cells, the levels of IL-22, a Th17-related-effector molecule, in the sera of patients with RA increase [32]. However, the IL-10 level in the sera of patients with RA in this study is decreased, which is contrary to other studies in which IL-10 is elevated in blood and SF [31, 33]. The IL-10 level in this study is consistent with another study, demonstrating that the IL-10 concentration in culture supernatants from PBMCs and SFMCs in patients with RA is lower than that in healthy controls [34]. The differences in immune cells and cytokines from patients with RA may be related to the difference in disease populations and stages. The dysregulation of these immune cells and cytokines may reflect inflammation rather than specific diseases.

Consistent with the view that Th17 and IL-17 have dominant pathogenic roles in RA [32], the Th17 cell frequency is increased in patients with high disease activity, whereas the Treg percentage is decreased. These results are consistent with the findings that active patients with RA have high expression of RORc mRNA and low expression of FoxP3 mRNA than that of inactive patients and healthy controls [35], because FoxP3 and RORc are the lineage-specific transcription factors of Treg and Th17 cells. In contrast to stable patients who have increased ILC1s and decreased ILC2s [11], patients with high disease activity have decreased ILC1s and increased ILC2s and ILC3s. Although ILC2s is thought to be involved in the resolution of inflammation in RA [24, 36], GM-CSF-producing ILC2s is reported to play a pathogenic role in the development of arthritis [37]. Takaki-Kuwahara A et al. revealed that the proportion of ILC1s in SF is negatively correlated with tender (TJC) and swollen (SJC) joint counts, but NKp44^+^ILC3s is positively correlated with TJC and SJC, suggesting that ILC3s aggravate the inflammation in patients with RA [10]. Activated ILCs are also important sources of inflammatory cytokines. High frequencies of Th17 and ILC3s are consistent with high levels of IL-17 in patients with RA and high disease activity. Furthermore, a low level of IL-10 is consistent with low frequencies of Treg cells in patients with RA and high disease activity. IL-10 is produced by Treg, Th1, Th2, and other cells [34]. This finding supports the notion that active patients may exhaust IL-10 due to continuous against inflammation [38]. Furthermore, these results are consistent with a previous study demonstrating that as inflammation increases, patients reveal low percentage of CD4^+^CD25^+^ Treg cells [38]. Thus, the imbalance in the Th17/Treg and ILC subsets involving the production of pro-/anti-inflammatory cytokines is associated with the development and/or progression of RA.

Patients positive for RF and/or ACPA are usually considered to be seropositive and considered to be correlated with high disease activity and poor clinical outcome [39]. RF^+^ and ACPA^+^ patients have increased percentages of ILC2s and ILC3s and decreased percentage of Treg and ILC1s relative to healthy controls. The above ILC subsets are not significantly different between RF^+^ and RF^−^ or ACPA^+^ and ACPA^−^ patients. Compared with HC, RF^+^, RF^−^, ACPA^+^, and ACPA^−^ patients have increased Th17 cells and IL-17A levels. Compared with HC, RF^+^ or ACPA^+^ patients have decreased Treg cells. However, the frequencies of Th17 and Treg cells show no statistical difference between RF^+^ and RF^−^ patients or between ACPA^+^ and ACPA^−^ patients. Although ACPA has been reported to stimulate macrophages to produce cytokines through the formation of immune complexes and participate in the pathogenesis of RA [40], we do not observe differences in the above immune cells and cytokines between ACPA^+^ and ACPA^−^ patients. No difference for the distribution of Th17 and Treg cells is observed between ACPA^+^ and ACPA^−^ patients, and this finding is consistent with a previous study [41]. However, Paulissen et al. reported that ACPA^+^ patients show high percentages of Th22, Th17.1, and CCR4/CXCR3 double-positive cells, which are gated from CCR6^+^ population and suggested that CCR6 + Th cell differentiates ACPA^+^ patients from ACPA^−^ patients [41]. Identifying the relationship between immune cell populations and autoantibodies may require identification markers. However, ACPA^+^ and RF^+^ as “seropositive” are commonly determined by anti-CCP2 IgG and IgM RF, respectively. The CCP2 test does not capture all ACPA because many proteins, such as citrullinated fibrinogen, collagen type II, tenascin C, α-enolase, vimentin, and histones, are recently described as ACPA targets [42]. Rönnelid and colleagues demonstrated that 16% of anti-CCP2-negative are ACPA^+^ in patients with RA by using a multiplex citrullinated peptide array [43]. Furthermore, the IgA isotype of RF and anti-CCP antibody has been found to be superior to IgM- or IgG-RF and anti-CCP response in mucosal inflammation and is closely associated with the development of RA [44–46]. Thus, Reed E et al. reported that ACPA^−^ or RF^−^ is not truly a seronegative subset in patients with RA [42]. ACPA fine specificities and IgA/IgG RF for anti-CCP2-/IgM RF − patients should be screened, which may contribute to early diagnosis and therapy for these “seronegative” patients [42]. Thus, immune cell and cytokine dysregulation should be analyzed on the basis of a broad spectrum of RF and ACPA serology. However, this study had limitations because the results were from a limited number of patients, so the sample size needs to be expanded for validation.

## Conclusion

In conclusion, we demonstrated that patients with RA have a variety of immune cell disorders, such as CD4^+^T, CD19^+^B, Th17, Treg, and ILC subsets, but these immune cell dysregulations may not be related to RF and ACPA autoantibodies. Further studies are necessary to unravel the roles of immune cell populations precisely in the development of RA by increased identification markers or screening a broad spectrum of RF and ACPA serology.

## Supplementary Information

Below is the link to the electronic supplementary material.

Supplementary file1 (PDF 330 KB)

## Data Availability

All data generated or analyzed during this study are included in this article.
