# Supplementary material for: Imbalance of Th17, Treg, and helper innate lymphoid cell in the peripheral blood of patients with rheumatoid arthritis
Source: Clin Rheumatol. 2022 Aug 4;41(12):3837–49. doi: 10.1007/s10067-022-06315-8 (PMC9652246; doi:10.1007/s10067-022-06315-8)

Supporting Information for

Imbalance of Th17, Treg, and Helper innate lymphoid cell in the peripheral blood of patients with rheumatoid arthritis

Ting Wang[1,2♦], Jinbing Rui[3♦], Wenqi Shan[1], Fei Xue[1], Dingqi Feng[1], Liyang Dong [1,4], Jiahui Mao[1], Yang Shu[1], Chaoming Mao[4], Xuefeng Wang[1,4]*

[1] Department of Central Laboratory, The Affiliated Hospital of Jiangsu University, Zhenjiang 212001, China;

[2] Department of Clinical Laboratory, The Affiliated Hospital of Jiangsu University, Zhenjiang 212001, China;

[3] Department of Rheumatology, The Affiliated Hospital of Jiangsu University, Zhenjiang 212001, China;

[4] Department of Nuclear Medicine and Institute of Oncology, The Affiliated Hospital of Jiangsu University, Zhenjiang 212001, China;

♦ These authors contributed equally to this work.

*Correspondence to: Xuefeng Wang, Department of Central Laboratory, The Affiliated Hospital of Jiangsu University, 438 Jiefang Road, Zhenjiang 212001, China. Phone: +86-0511-8502-1135; E-mail: xuefengwang@ujs.edu.cn; ORCID: 0000-0002-5980-6530

Table S1 Characteristics of patients with RA and healthy controls

Demographics	RA patients (n = 86)	Healthy (n = 50)	p value
Age (years) - mean ± SEM	55.43 ± 1.46	51.46 ± 1.40	0.0721
Sex - female/male	12/74	10/40	>0.999
RF - median (range)	54.90 (23.03 - 200.3)	-	-
ACPA - median (range)	234.10 (37.18 - 800)	-	-
ESR (mm/h) - median (range)	25.00 (12.50 - 51.00)	4.20 (2.45 - 6.90)	<0.0001
CRP (mg/L) - median (range)	6.30 (2.25 - 16.35)	0.50 (0.50 - 1.23)	<0.0001
Disease duration (years) - median (range)	6.00 (1.25 - 10.00)	-	-
DAS28 - median (range)	3.12 (2.35 - 4.11)	-	-

RF, rheumatoid factor; *ACPA*, anti-citrullinated protein antibodies; *ESR*, erythrocyte sedimentation rate; *CRP*, C-reactive protein; *DAS28*, disease activity score in 28 joints.

Figure S1 Gating strategies for the identification of ILCs. Isolated cells were pre-gated on LIN$^-$(CD3$^-$CD19$^-$CD56$^-$) cells, then total ILCs (CD3$^-$CD19$^-$CD56$^-$CD127$^+$), ILC1s (LIN$^-$CD294$^-$CD117$^-$), ILC2s (LIN$^-$CD294$^+$), and ILC3s (LIN$^-$CD294$^-$CD117$^+$) in a representative sample.

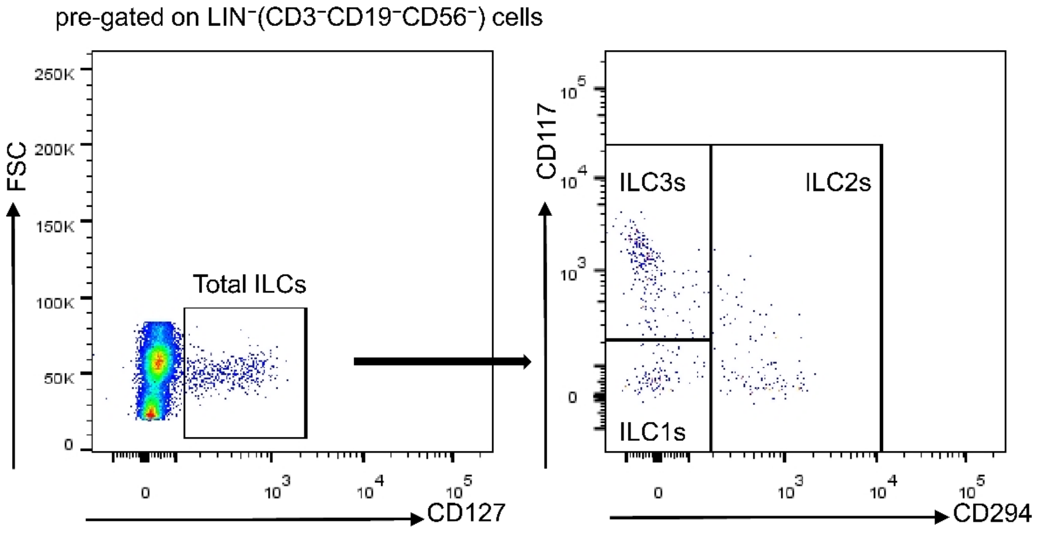

Figure S2 Differences in ESR, CRP, and RF among HC and RA patients with different disease activity status. (a)ESR, (b) CRP, (c) RF. Results are presented as mean ± SEM. * $P < 0.05$, ** $P < 0.01$.

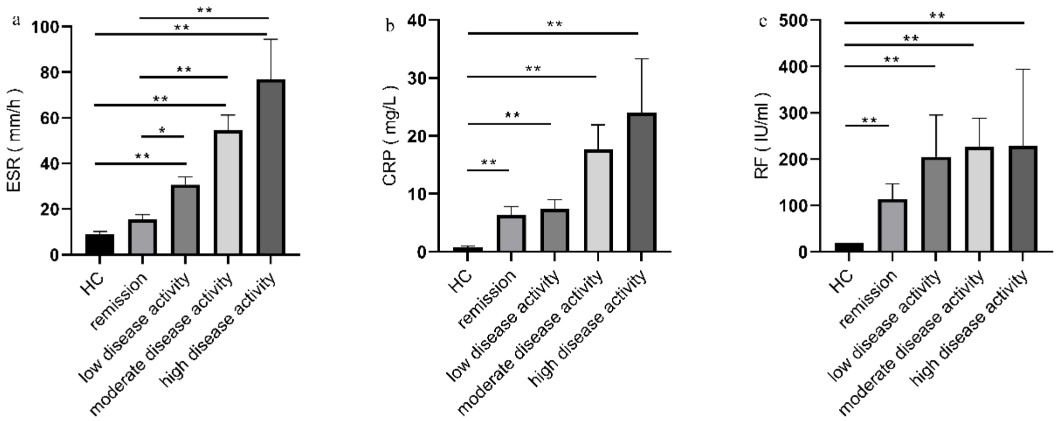

Figure S3. Cytokine analysis in sera by ELISA in HC, RF$^-$, and RF$^+$ RA patients.

(a) IFN-γ, (b) IL-4, (c) IL-10, (d) IL-17A, (e) IL-22, and (f) IL-33. Results are presented as mean ± SEM. * $P < 0.05$, **** $P < 0.0001$.

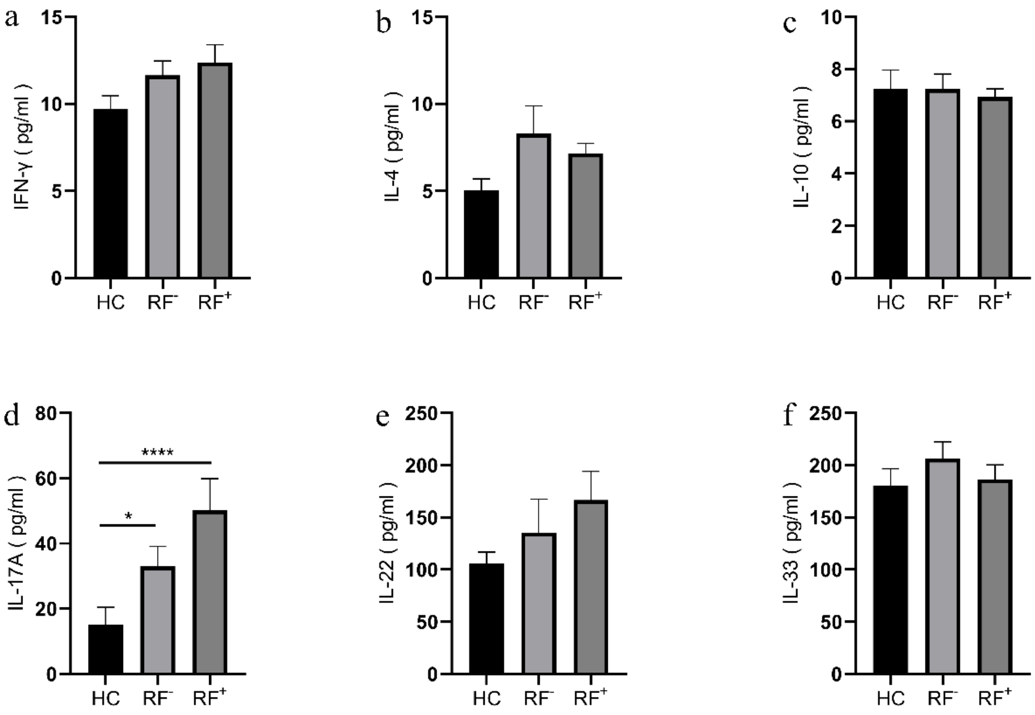

Figure S4. Cytokine analysis in sera by ELISA in HC, ACPA⁻, and ACPA⁺ RA patients.

(a) IFN-γ, (b) IL-4, (c) IL-10, (d) IL-17A, (e) IL-22, and (f) IL-33. Results are presented as mean ± SEM. * *P* < 0.05, **** *P* < 0.0001.

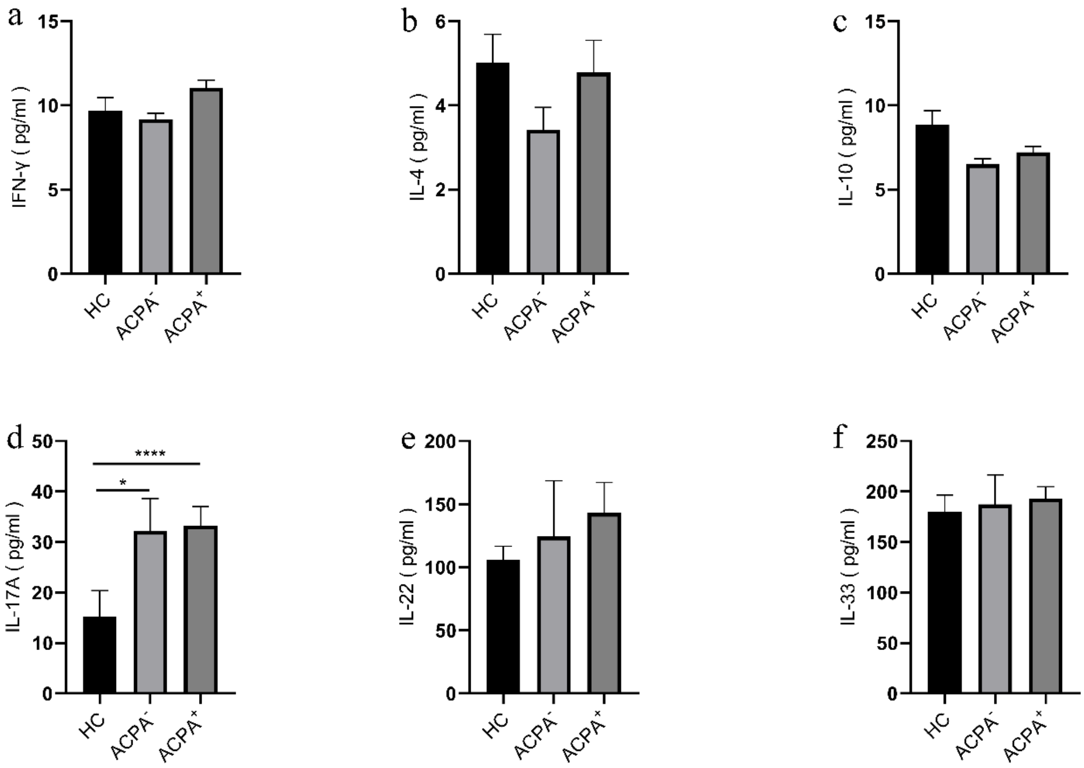

Supplement: Supplementary file 1 — Supplementary file1 (PDF 330 KB) [file 10067_2022_6315_MOESM1_ESM.pdf]
